# Establishment and characterization of immortalized Gli-null mouse embryonic fibroblast cell lines

**DOI:** 10.1186/1471-2121-9-49

**Published:** 2008-09-13

**Authors:** Robert J Lipinski, Maarten F Bijlsma, Jerry J Gipp, David J Podhaizer, Wade Bushman

**Affiliations:** 1 Molecular and Environmental Toxicology Center, School of Medicine and Public Health, University of Wisconsin-Madison, Madison, WI, USA; 2 Department of Surgery, School of Medicine and Public Health, University of Wisconsin-Madison, Madison, WI, USA; 3 Center for Experimental and Molecular Medicine, Academic Medical Center, Amsterdam, The Netherlands

## Abstract

**Background:**

Hedgehog (Hh) signaling is a conserved morphogenetic pathway which plays critical roles in embryonic development, with emerging evidence also supporting a role in healing and repair processes and tumorigenesis. The Gli family of transcription factors (Gli1, 2 and 3) mediate the Hedgehog morphogenetic signal by regulating the expression of downstream target genes. We previously characterized the individual and cooperative roles of the Gli proteins in Hh target gene regulation using a battery of primary embryonic fibroblasts from Gli null mice.

**Results:**

Here, we describe the establishment of spontaneously immortalized mouse embryonic fibroblast (iMEF) cell lines lacking single and multiple Gli genes. These non-clonal cell lines recapitulate the unique ligand mediated transcriptional response of primary MEFs. While loss of Gli1 had no effect on target gene induction, Gli2 null cells demonstrated reduced target gene induction while Gli3 null cells exhibited elevated basal and ligand-induced expression. Target gene response in *Gli1*^-/-^*2*^-/- ^iMEFs was severely reduced while *Gli2*^-/-^*3*^-/- ^iMEFs were incapable of ligand-induced transcriptional response. However, we found that both *Gli1*^-/-^*2*^-/- ^and *Gli2*^-/-^*3*^-/- ^iMEFs exhibited robust leukotriene synthesis-dependent migration responses to Hh ligand, demonstrating that this response is not transcriptionally-dependent.

**Conclusion:**

This study provides fundamental characterizations of the transcriptional and non-transcriptional Hh responsiveness of a battery of Gli-null iMEFs. Moving forward, these cell lines should prove a valuable tool set to study the unique functional regulation of the Gli proteins in a Hh-responsive cell-type.

## Background

The Hedgehog (Hh) signaling pathway is a critical regulator of diverse biological processes including developmental patterning and organogenesis. The pathway is initiated upon Hh ligand binding to the transmembrane receptor Patched (Ptc1). This relieves the Ptc1-mediated suppression of Smoothened (Smo), triggering a complex downstream signal cascade [Reviewed in [1]]. *Gli1 *and *Ptc1 *are conserved Hh target genes and their expression levels are considered reliable indicators of pathway activity. Most biological effects of Hh signaling appear to be mediated through transcriptional regulation of Hh target genes, although a non-transcriptional response was recently identified [2,3].

Null mouse models have been critical in determining the role of Hh signaling in the growth and morphogenesis of tissues and organs. These models have also proved valuable in gleaning the function of individual Hh signal mediators in pathway regulation. In cell-based assays, *Gli1 *over-expression has been found to induce Hh target gene expression. The finding that *Gli1*^-/- ^mice develop normally, [4] however, infers that Gli1 function is dispensable for normal development. *Gli2*^-/- ^mice exhibit neural tube defects and demonstrate diminished Hh target gene expression in several tissues [5-7]. This supports findings from cell-based assays [8] that Gli2 functions as a critical target gene activator. Increased target gene expression in tissues derived from *Gli3 *null mice relative to tissues from wild type mice [9,10] suggests that Gli3 functions to repress transcription.

Numerous studies have utilized transgenic MEFs to investigate diverse gene and protein properties. However, the experimental utility of primary cells is limited by a finite propagation and culture period. We showed previously that mouse embryonic fibroblasts (MEFs) from Gli null mice provide a tractable cell-based system in which to quantitatively examine the regulation of Hh target gene expression by the Gli transcription factors [11]. We now describe the generation of immortalized Gli null MEFs (iMEFs) and characterize their transcriptional and migratory response to Hh ligand stimulation.

## Methods

### Animals

This work was conducted with the approval of the University of Wisconsin Animal Care and Use Committee. *Gli1*^*zfd *^and *Gli2*^*zfd *^mice were generously provided by Alexandra Joyner and maintained on an outbred CD-1 background. *Gli3*^*Xtj *^mice were obtained from Jackson Laboratories (Bar Harbor, ME) and were maintained on a C57/C3H background. Primary MEFs [11] were derived from crosses of *Gli1*^*zfd *^and *Gli2*^*zfd *^transgenic mice and *Gli3*^*Xtj *^mutant mice. *Gli1*^*zfd *^and *Gli2*^*zfd *^transgenic mice were produced by homologous recombination replacing exons 2–5 and 3–5, respectively with neo cassettes [4,12]. *Gli3*^*Xtj *^mutant mice from Jackson Laboratories (Bar Harbor, ME) lack *Gli3 *expression due to a deletion mutation in the 3' end of the gene [13].

### Cell immortalization

Primary MEFs were grown as described previously (Lipinski et al., 2006) in 10% fetal calf serum (FCS) DMEM [with L-glutamine, 4.5 g/L glucose, without sodium pyruvate] with 1% Pen/Strep and propagated following the 3T3 protocol for spontaneous immortalization [14]. 3.0 × 10^5 ^cells in 4.0 mls media were plated in 60 mm plates and passed at three day intervals. After 8–12 passes, proliferation rates decreased and cells were allowed to grow to confluence before subsequent passing. After 15–25 passes, proliferation rates increased, suggesting spontaneous immortalization. Following, cells were grown for an additional 10–12 passes to ensure stable immortalization. The absence of expression of *Gli1*, *Gli2*, and *Gli3 *in corresponding null iMEF cell lines was confirmed by Real Time-RT-PCR of isolated cDNA [11] as well as standard genotyping of genomic DNA [4,12].

### Generation of stable over-expresser cell lines by retroviral gene delivery

A pIRES shuttle vector carrying coding sequences for *hShh*, *hGli1*, *ΔNmGli2*, hSmo* and independently translated GFP [15] was used to retrovirally infect WT iMEFs. iMEFs were plated at subconfluence in DMEM with 10% FCS 100 mm plates. Cells were then incubated with viral-conditioned media at 4°C for 6 hrs. Following a 72 hr propagation period, GFP-sorting was used to isolate over-expressing populations.

### Cell treatment and Real Time RT-PCR

iMEFs were plated in Multiwell Primaria™ 24 well plates (Falcon, Franklin Lakes, NJ) at 2.0 × 10^5 ^cells per well in 400 μl media. Cells were allowed to attach overnight and media were replaced with DMEM containing 1% FCS ± 1 nM octylated Shh peptide (Curis/Genentech). At 24 hrs RNA was harvested and gene expression was determined by real time RT-PCR as described, [11] using gene specific primers as listed: *GAPDH*: 5'-AGCCTCGTCCCG TAGACAAAAT-3' and 5'-CCGTGAGTG GAGTCATACTGGA-3', *Ptc1*: 5'-CTCTGGAGCAGATTTCCAAGG-3' and 5'-TGCCGCAGTTCTTTTGAATG-3', *Gli1*: 5'-GGAAGTCCTATTCACGCCTTGA-3' and 5'-CAACCTTCTTGCTCACACATG TAAG-3', *Gli2*: 5'-CCTTCTCCAATGCCT CAGAC-3' and 5'-GGGGTCTGTGTACCT CTTGG-3', *Gli3*: 5'-AGCCCAAGTATTATT CAGAACCTTTC-3' and 5'-ATGGATAGG GATTGGGAATGG-3'.

### Migration assays

Cells were grown to 70% confluence in 6-well plates and labeled with 10 μM CellTracker Green (Invitrogen) in serum-free medium for 1 hr. The dye was fixed by adding 10% FCS for 1 h, and subsequently cells were washed and detached with 5 mM ethylenediaminetetraacetic acid (EDTA) in PBS. After complete detachment, cells were resuspended in serum-free medium, pipetted through a 70 μM cell strainer (BD Falcon, Franklin Lakes, NJ), and 100 μl suspension was transferred to 8 μM pore size HTS FluoroBlok Cell Culture Inserts from BD Falcon which were inserted in fitting 24-well plates. In the bottom wells, 600 μl medium was supplemented with indicated chemoattractant. Promptly, fluorescence values representing the number of cells on the bottom side of the insert were read four times every two minutes on a Series 4000 CytoFluor Multi-Well Plate Reader (Perseptive Biosystems, Framingham, MA). The raw fluorescence data were corrected for background fluorescence. No-attractant controls were subtracted at each measured time point to correct for any effects not due to active migration to the chosen attractant. Migration start points were set to zero. For comparison of the different cell lines from multiple experiments, total migration of wild type cells was set to one. For migration assays, Shh peptide (R&D Systems) was used at given concentrations. Preincubation with inhibitors was performed during 10 minutes following detachment and inhibitors were also added to the bottom wells to exclude chemorepellent artifacts. Transfection of iMEFs with *SuFu *overexpression construct (a kind gift of Dr. Toftgård) was performed with Effectene transfection reagent (Qiagen, Hilden, Germany) according to manufacturer's recommendations 16 h before start of migration assay. Western blot analysis revealed a 10-fold increase in SuFu levels following transfection (not shown).

## Results and discussion

### MEF immortalization, morphological characterization, and ploidy analysis

**Figure 1:**
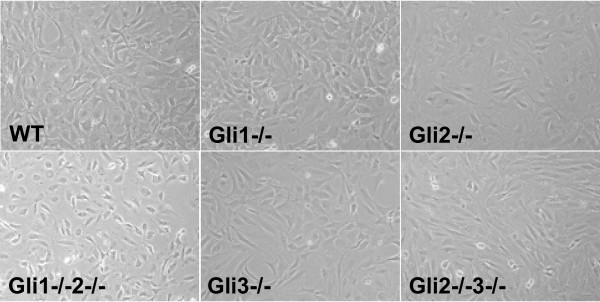
**Gli-null iMEF morphology in monolayer cell culture**. Indicated iMEFs were grown to confluence in monolayer culture and imaged at 40× magnification.

*Gli3*^+/+ ^(WT), *Gli1*^-/-^, *Gli2*^-/-^, *Gli3*^-/-^, *Gli1*^-/-^*2*^-/-^, and *Gli2*^-/-^*3*^-/- ^primary MEFs were propagated by described 3T3 protocols for spontaneous immortalization [14]. Each non-clonal immortalized cell line demonstrated a fibroblast-like morphological appearance in monolayer culture although individual lines exhibited subtle morphological differences (Figure 1). Each iMEF line was determined to be tetraploid by flow cytometry analysis (data not shown).

### Characterization of iMEF transcriptional Hh responsiveness

**Figure 2:**
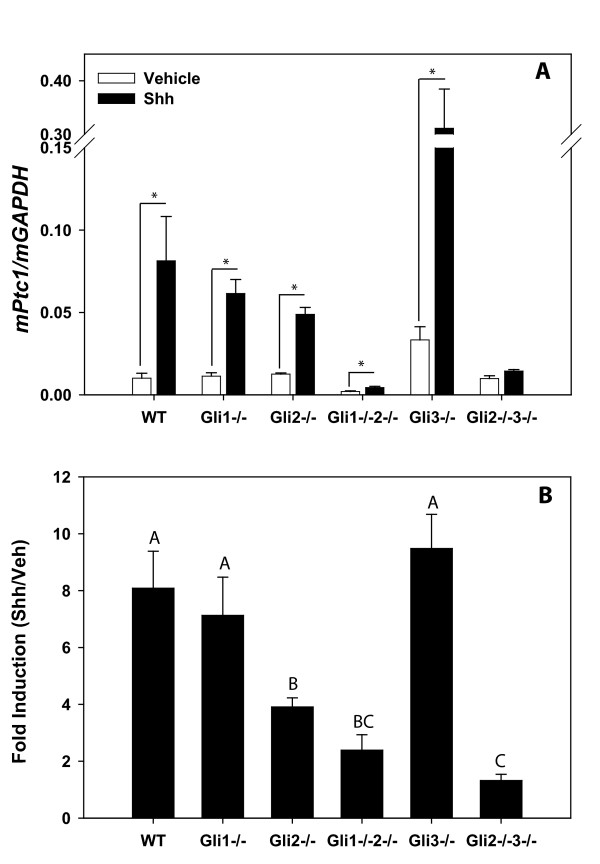
**Transcriptional Hh-responsiveness of generated iMEFs**. Indicated iMEFs were plated at confluence and treated ± Shh ligand. After 24 hrs, expression of *Ptc1 *was determined by Real-Time RT-PCR. A. Basal and Shh-induced expression of *Ptc1*. Values represent the mean ± SEM of 3–5 replicate experiments, * indicates P ≤ 0.05 (paired t-test). B. *Ptc1 *expression plotted as fold induction (Shh/Veh). Values represent the mean ± SEM of three replicate experiments. The letters above the bars denote the groups produced by the ANOVA pair-wise differences. Genotypes sharing a letter are not statistically significant at p ≤ 0.05 (Fisher's LSD).

iMEFs were treated ± Shh ligand, and Hh target gene expression was determined by real time RT-PCR. Figure 2A shows the expression of reliable Hh target gene *Ptc1 *following stimulation with Shh or vehicle and Figure 2B shows the fold induction (Shh/Veh) of *Ptc1 *expression. *Gli3*^-/- ^iMEFs demonstrated elevated basal and Shh-induced expression of *Ptc1 *(p = 0.03 and p = 0.02 respectively) relative to WT cells. Shh ligand stimulation induced *Ptc1 *expression in each iMEF line except that lacking expression of both *Gli2 *and *Gli3*, which are essential for a transcriptional Hh response. While loss of Gli1 alone had no effect on target gene expression, *Ptc1 *induction was reduced in both *Gli2*^-/- ^and *Gli1*^-/-^*2*^-/- ^iMEFs relative to WT.

### Characterization of iMEF non-transcriptional Hh responsiveness

**Figure 3:**
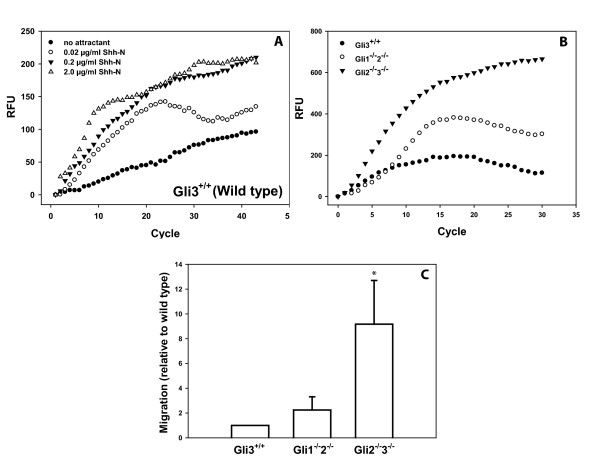
**Non-transcriptional Hh-responsiveness of the generated iMEFs**. A. Example of a migration assay using wild type (*Gli3*^+/+^) iMEFs in a Transwell system with varying concentrations of Shh as chemoattractant. Fluorescence was read every two minutes and expressed as relative fluorescence unit (RFU). B. Example of a migration assay using wild type (*Gli3*^+/+^), *Gli1*^-/-^*2*^-/- ^and *Gli2*^-/-^*3*^-/- ^iMEFs with 2 μg/ml Shh as chemoattractant. No-attractant condition was subtracted and migration starting points were set to t = 0. Robust migration was observed for each cell line. C. Total migration data from several experiments as performed for B, pooled and expressed as fraction of wild type iMEF migration (*Gli3*^+/+^, set to 1, n = 5). To determine whether the migration response was significantly different relative to wild type, a 95% confidence interval was calculated based on the mean and standard deviation of the observations. Reported significant differences thus have a P value of < 0.05.

While Hh signaling effects are thought to be exerted primarily through transcriptional regulation, a novel pathway was recently identified which is Smo-dependent but does not require transcription [2,3]. This alternative pathway triggers cytoskeletal rearrangement, driving a cellular migratory response toward Hh ligand. When activation of this pathway was investigated in wild type iMEF cells, a dose-dependent migratory response to recombinant Shh was observed (Figure 3A). In the absence of Shh ligand (no-attractant control), a low level of baseline migration was observed and subsequently subtracted from the migratory responses in all other experiments.

The generated Gli null iMEFs provide a valuable tool to assess transcription factor dependence of specific biological responses. When *Gli3*^+/+ ^(WT), *Gli1*^-/-^*2*^-/-^, and *Gli2*^-/-^*3*^-/- ^iMEF cells were allowed to migrate to 2 μg/ml Shh peptide, migration was observed for each genotype (Figure 3B). Remarkably, increased migration to Shh was observed for the null cells (*Gli1*^-/-^*2*^-/- ^and *Gli2*^-/-^*3*^-/-^) that was inversely correlated with their respective transcriptional Hh-responsiveness (Figure 3C). This inverse correlation may be explained by competition for shared pathway components between the two different signal transduction mechanisms but no data to suggest such a competition have so far been presented.

**Figure 4:**
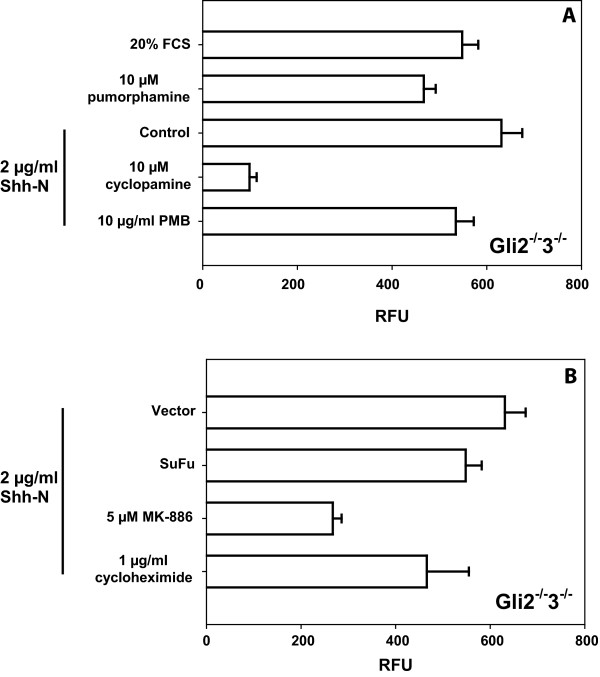
**Hedgehog pathway specificity in migratory response of Gli2^-/-^3^-/- ^iMEFs**. A. Migration of *Gli2*^-/-^*3*^-/- ^iMEFs was performed using the Smo agonist purmorphamine or 20% FCS as attractants as well as 2 μg/ml Shh in the presence of no inhibitor (control), the Smo antagonist cyclopamine or the LPS inhibitor PMB. Preincubation time with inhibitors following detachment was 10 minutes. B. Migration responses of *Gli2*^-/-^*3*^-/- ^iMEFs transfected with a SuFu or control vector or preincubated with the leukotriene synthesis inhibitor MK-886 or translation inhibitor, cycloheximide. No-attractant condition was subtracted and migration starting points were set to t = 0.

To confirm that the observed migration of *Gli2*^-/-^*3*^-/- ^iMEFs to Shh peptide is Hh-pathway specific, we used the Smo agonist purmorphamine as chemoattractant. Robust migration was observed that was comparable in magnitude to migration to Shh as well as the positive control, FCS (Figure 4A). Conversely, treatment with the Smo inhibitor cyclopamine abrogated the migration response to Shh peptide. These data indicate that the migratory response of *Gli2*^-/-^*3*^-/- ^iMEFs is Smoothened dependent and thus Hh-pathway dependent. An artifactual response to endotoxin contamination of Shh peptide [16] was excluded by demonstrating the inability of the lipopolysaccharide (LPS) inhibitor polymixin B (PMB) to reduce the cellular migratory response to Shh peptide.

While the Hh-inhibitory protein suppressor of fused (SuFu) mitigates the Gli-mediated Hh transcriptional response, it appears to have no effect on migratory response [2]. Accordingly, when SuFu was overexpressed in *Gli2*^-/-^*3*^-/- ^iMEFs, Shh-induced migration was not changed (Figure 4B). The translation independence of the migratory response was confirmed by demonstrating the ineffectiveness of cycloheximide in altering the migration response to Shh peptide.

To confirm the previously demonstrated requirement for intact leukotriene synthesis machinery in the migratory response to Shh [2,3], we used the lipoxygenase inhibitor MK-886 to block leukotriene production. When *Gli2*^-/-^*3*^-/- ^iMEFs were preincubated with 5 μM MK-886 migration to Shh was markedly reduced, indicating that in cells without a functional transcriptional Hh signaling pathway, leukotriene synthesis is required for Shh-mediated migration (Figure 4B).

As several studies have demonstrated that either Gli2 and Gli3 are required for the Hh signaling transcriptional response [9,11,17], the Hh-induced migration of *Gli2*^-/-^*3*^-/- ^cells is affirmative evidence that the migratory response is independent of Gli transcription factor activity. Also important for this study, the observed migration data indicate that the iMEFs have functional Hh-sensing machinery and that the diminished Hh-responsiveness of *Gli1*^-/-^*2*^-/- ^and *Gli2*^-/-^*3*^-/- ^iMEFs is due to the absence of Gli proteins, rather than ablation of the Ptch1/Smo receptor pair or other artifacts.

### Stable over-expression of Hh components drives constitutive pathway activation

**Figure 5:**
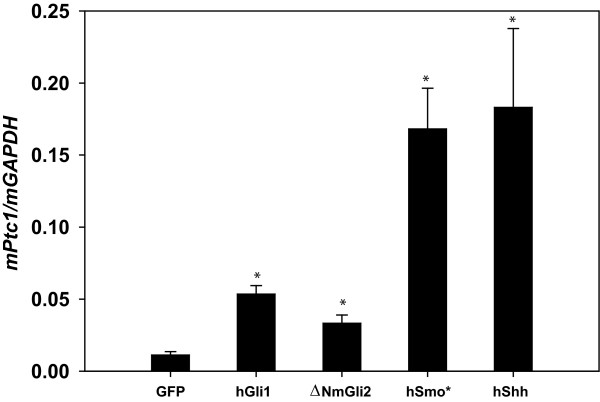
**Stable over-expression of several Hh components drives constitutive pathway activity**. WT iMEFs were infected with retrovirus encoding, *hShh-GFP*, *hGli1-GFP*, *ΔNmGli2-GFP*, *hSmo-GFP *or an empty *GFP *IRIS vector. Stable over-expresser cell lines produced by GFP sorting were plated at confluence. Following 24 hrs, expression of *Ptc1 *was determined by Real-Time RT-PCR. Values represent the mean ± SEM of three replicate experiments, * indicates P ≤ 0.05 (paired t-test) vs. GFP iMEFs.

Immortalized cells allow for retroviral-mediated, stable expression of vectors for gene-knockdown or over-expression. We generated WT iMEFs with stable over-expression of several pathway components and assessed pathway activity by measuring the expression of *Ptc1*, a reliable Hh target gene. We found that iMEFs over-expressing *hShh*, *hGli1*, or constitutively active forms of *mGli2 *(*ΔNmGli2*) or *hSmo *(Smo*) demonstrated increased pathway activity relative to iMEFs expressing only GFP (Figure 5). This demonstrates that over-expression of pathway components at multiple levels including ligand and transcription factor is sufficient to drive constitutive pathway activity.

## Conclusion

The full complement of Gli genes in most Hh ligand-responsive cell models mitigates their utility in investigations of molecular regulation and biological activity of the individual Gli transcription factors. Here we demonstrated the unique transcriptional and non-transcriptional responses of a battery of Gli-null iMEFs. Moving forward, these cell lines should prove a useful tool in a wide range of the Hh signaling field and have already been distributed to several investigators for a wide range of purposes including; studies of transcriptional co-regulators and Gli-binding partners; chemical pathway inhibitor site of action studies; anti-Gli antibody specificity studies; and several studies of Gli dependence in specific Hh-related biological function.

## Authors' contributions

RJL and DJP isolated primary cells, established immortalized cell lines and confirmed proper genotypes. JJG performed transcriptional response assays and MFB performed migration assays. Each was responsible for data acquisition and analysis. WB participated in the design and interpretation of experiments. All authors contributed to preparation of the manuscript and have approved the final form.
